# PMA inhibits endothelial cell migration through activating the PKC-δ/Syk/NF-κB-mediated up-regulation of Thy-1

**DOI:** 10.1038/s41598-018-34548-8

**Published:** 2018-11-02

**Authors:** Heng-Ching Wen, Yen Nien Huo, Chih-Ming Chou, Wen-Sen Lee

**Affiliations:** 1 Graduate Institute of Medical Sciences, College of Medicine, Taipei Medical University, Taipei, 110 Taiwan; 2 Department of Biochemistry and Molecular Cell Biology, School of Medicine, College of Medicine, Taipei Medical University, Taipei, 110 Taiwan; 3 Department of Physiology, School of Medicine, College of Medicine, Taipei Medical University, Taipei, 110 Taiwan; 4 Cancer Research Center, Taipei Medical University Hospital, Taipei, 110 Taiwan

## Abstract

We previously showed that overexpression of Thy-1 inhibited and knock-down of Thy-1 enhanced endothelial cell migration. Here, we used phorbol-12-myristate-13-acetate (PMA) as an inducer for Thy-1 expression to investigate molecular mechanisms underlying Thy-1 up-regulation. Our data showed that increased levels of Thy-1 mRNA and protein in endothelial cells were observed at 14–18 hours and 20–28 hours after PMA treatment, respectively. Treatment with PMA for 32 hours induced Thy-1 up-regulation and inhibited capillary-like tube formation and endothelial cell migration. These effects were abolished by Röttlerin (a PKC-δ inhibitor), but not Gö6976 (a PKC-α/β inhibitor). Moreover, pre-treatment with Bay 61–3606 (a Syk inhibitor) or Bay 11-7082 (a NF-κB inhibitor) abolished the PMA-induced Thy-1 up-regulation and migration inhibition in endothelial cells. Using the zebrafish model, we showed that PMA up-regulated Thy-1 and inhibited angiogenesis through the PKC-δ-mediated pathway. Surprisingly, we found that short-term (8–10 hours) PMA treatment enhanced endothelial cell migration. However, this effect was not observed in PMA-treated Thy-1-overexpressed endothelial cells. Taken together, our results suggest that PMA initially enhanced endothelial cell migration, subsequently activating the PKC-δ/Syk/NF-κB-mediated pathway to up-regulate Thy-1, which in turn inhibited endothelial cell migration. Our results also suggest that Thy-1 might play a role in termination of angiogenesis.

## Introduction

Angiogenesis, generation of new blood vessels from pre-existing vessels, is a major process through which the vascular expands during embryonic development, the formation of corpus luteum, organ growth, wound healing, and tissue regeneration^1^. Angiogenesis is characterized by the endothelial cells grown toward the angiogenic stimulus, and it usually occurs in the poorly perfused tissues at the hypoxia condition to satisfy the metabolic requirements^2^. The process of angiogenesis involves consecutive steps, including degradation of the basement membrane, endothelial cell migration and proliferation, loop formation, and vascular stabilization^3^. Proliferation and migration of vascular endothelial cells are two critical steps of angiogenic process. Although angiogenesis plays an essential role in physiologic processes, the dysregulated angiogenesis contributes to the pathogenesis of many disorders, including psoriasis, ocular neovascularization, arthritis, and cancer^1,4,5^. Therefore, understanding the mechanism of angiogenesis regulation may provide new insight into angiotherapy.

The initiation and termination of angiogenesis are thought to be strictly controlled by the balance between positive and negative regulators^6^. Normally, endothelial cells maintain in a quiescent state that is regulated by endogenous angiogenesis inhibitors over angiogenic stimuli in a healthy adult organism^7,8^. However, in pathological conditions, especially in the tumor, angiogenesis is stimulated not only by overexpression of proangiogenic factors but also by down-regulation of inhibitory factors. The initiation of angiogenesis has been intensively investigated; however, very little is known about the control of termination of angiogenesis^8^.

Thy-1, a 25–37 kDa glycosylphosphatidylinositol (GPI)-anchored cell surface protein, has been recognized to be important for immunologic functions, such as T cell activation and proliferation, and thymocyte differentiation in mouse^9,10^. Moreover, Thy-1 also has a variety of non-immunological functions, including wound healing, cell adhesion, migration, proliferation and apoptosis, and cell-cell interaction^11^. In addition to thymocytes and T-cells, Thy-1 has been also found to be expressed in several cell types, such as activated endothelial cells, vascular pericytes, neurons, mesenchymal cells, and fibroblasts^12^. Previously, we demonstrated that Thy-1 can serve as a novel marker of adult, but not embryonic, angiogenesis^13^. We also demonstrated that overexpression of Thy-1 inhibited vascular endothelial cell migration and capillary-like tube formation through reducing the RhoA activity^14^. However, the molecular mechanism underlying Thy-1 up-regulation in vascular endothelial cells is still not clear.

Previous *in vitro* studies showed that phorbol-12-myristate-13-acetate (PMA) can up-regulate Thy-1 expression in human dermal microvascular endothelial cells (HDMECs)^15^. We also showed that PMA can reduce the endothelial migration, and this effect was abolished by knock-down of Thy-1 expression using siRNA technique^14^. Accordingly, we used PMA as an inducer of Thy-1 expression to investigate the regulation of Thy-1 expression in vascular endothelial cells and the effect of PMA on angiogenesis. The findings of the present study will provide important insights into the mechanism by which Thy-1 expression is regulated. Understanding the molecular mechanism of Thy-1 induction may provide novel therapeutic strategies for treatment of angiogenesis-related diseases.

## Results

### Effects of PMA on Thy-1 expression in endothelial cells

**Figure 1:**
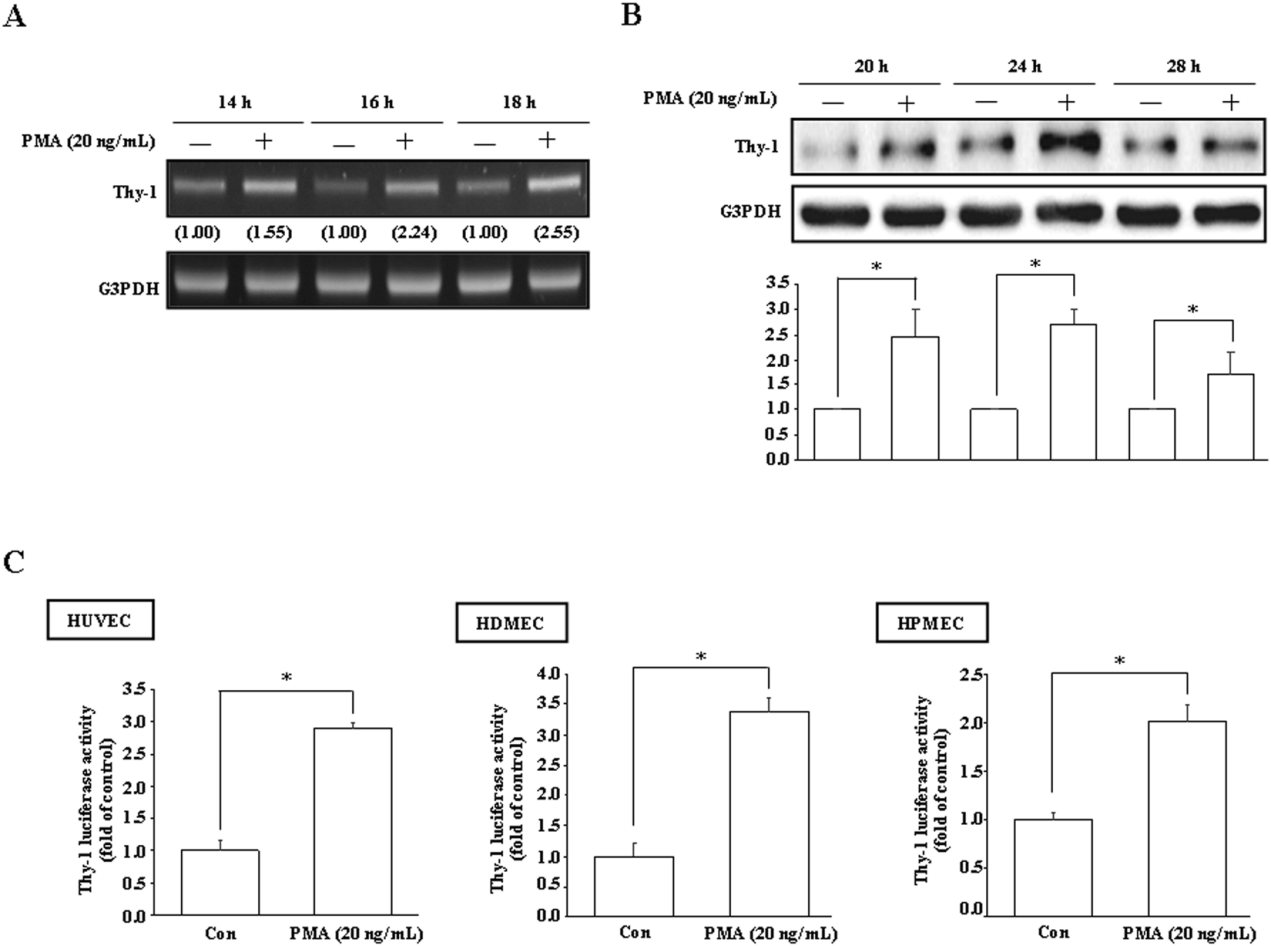
PMA up-regulates Thy-1 expression in vascular endothelial cells. PMA (20 ng/mL) increased the levels of Thy-1 mRNA (**A**) and protein (**B**) in a time-dependent manner detected by RT-PCR and Western blot analysis, respectively. Values shown in parentheses represent the quantified results adjusted with G3PDH and expressed as ratio over control. (**C**) PMA (20 ng/mL) increased the Thy-1 promoter activity in HUVECs, HDMECs, and HPMECs. The luciferase activity was normalized by Renilla luciferase expression. In (**B**) and (**C**), n = 3. The cropped blot was shown. The entire gel picture of 1B (Thy-1) was shown in the Supplemental Fig. S1. *p < 0.05, different from corresponding control. Con, control.

To study the molecular mechanism underlying Thy-1 induction, we used PMA, which has been reported to be able to increase the levels of Thy-1 mRNA and protein^15^, as a stimulator for Thy-1 expression. Initially, RT-PCR and Western blot analyses were conducted to examine the effect of PMA on Thy-1 expression. Treatment with PMA (20 ng/mL) time-dependently increased the levels of Thy-1 mRNA and protein in HUVECs (Fig. 1A,B). Moreover, PMA significantly increased the Thy-1 promoter activity in HUVECs, HDMECs, and human pulmonary microvascular endothelial cell line (HPMECs) (Fig. 1C).

### PKC-δ activation is involved in the PMA-induced Thy-1 up-regulation and migration inhibition in endothelial cells

**Figure 2:**
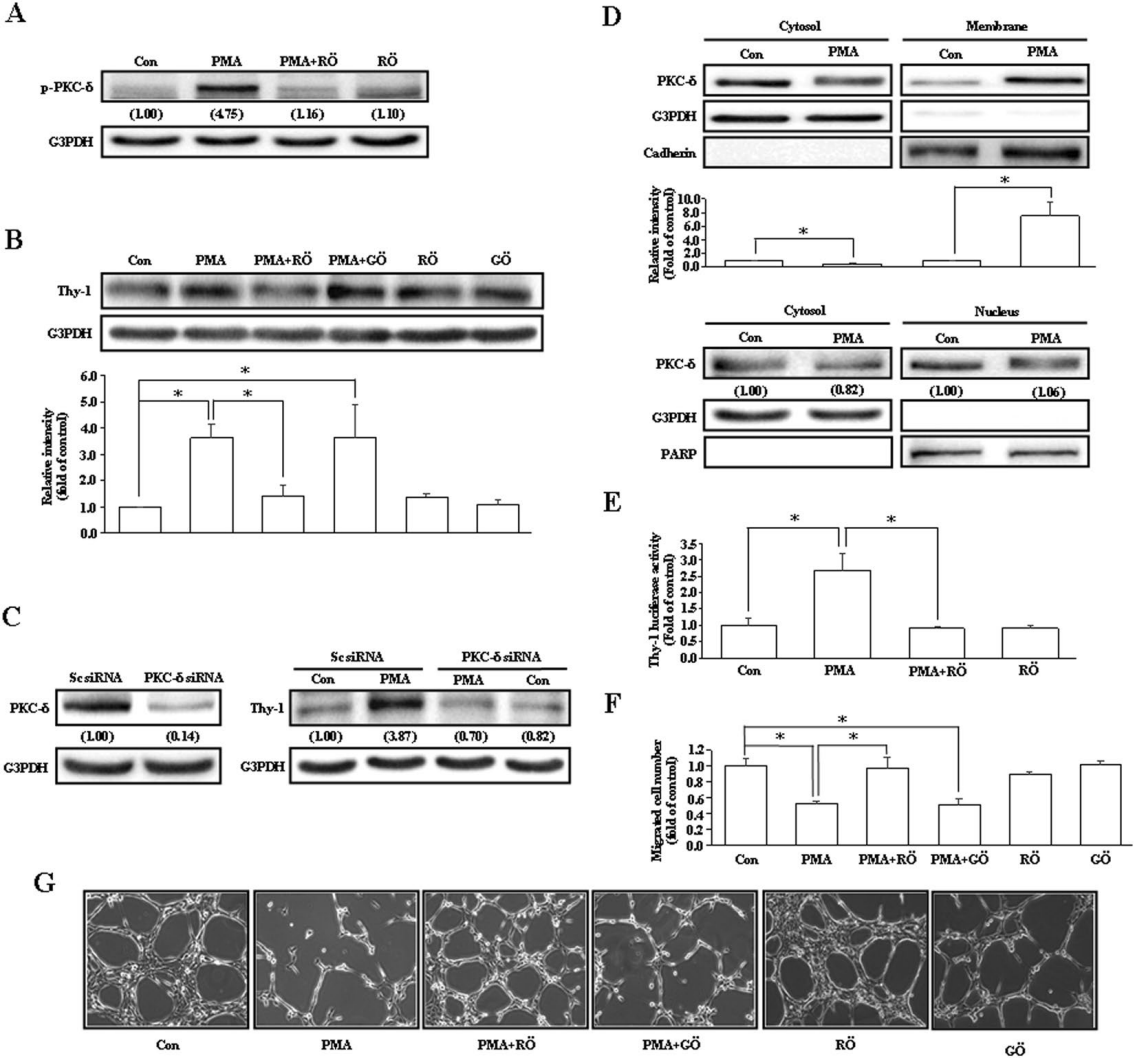
PKC-δ is involved in PMA-induced increases of Thy-1 protein. Pre-treatment with Röttlerin (5 μM), a PKC-δ inhibitor, abolished the PMA (20 ng/mL)-induced PKC-δ activation (**A**) and Thy-1 up-regulation (**B**). However, the PMA-induced Thy-1 up-regulation was not significantly affected by pre-treatment with Gö6976 (200 nM), a PKC-α/β inhibitor. In (A), values shown in parentheses represent the quantified results adjusted with G3PDH. In (**B**), n = 3. (**C**) Transfection with PKC-δ siRNA abolished the PMA-increased the level of Thy-1 protein. Values shown in parentheses represent the quantified results adjusted with G3PDH and expressed as ratio over control. (**D**) PMA increased PKC-δ membrane translocation (top panel), but did not affect the level of nuclear PKC-δ (bottom panel). Cadherin, G3PDH and PARP were used as a plasma membrane, cytosolic, and nuclear protein marker, respectively, to confirm the purities of isolation and to verify equivalent sample loading. In top panel, n = 4. In bottom panel, values shown in parentheses represent the quantified results adjusted with G3PDH or PARP and expressed as ratio over control. (**E**) Pre-treatment with Röttlerin abolished PMA-increased the Thy-1 promoter activity. The luciferase activity was normalized by Renilla luciferase expression. n = 3. (**F**) Röttlerin, but not Gö6976, prevented the PMA-induced migration inhibition. n = 4. (**G**) PMA inhibited the capillary-like tube formation. Pre-treatment with Röttlerin, but not Gö6976, prevented the PMA-inhibited capillary-like tube formation. The cell was pre-treated with Röttlerin or Gö6976 for 1 hour followed by PMA for an additional 24 hours, and then processed for Western blot analyses, Luciferase assay, wound-healing assay, or capillary-like tube formation assay. For PKC-δ siRNA transfection, the cell was transfected with PKC-δ siRNA (50 nM) or scramble siRNA for 40 hours followed by PMA treatment for an additional 24 hours, and then processed for Western blot analysis. The gels have been run in the same experimental conditions and the cropped blots were shown. The entire gel pictures of 2A (p-PKC-δ), 2B (Thy-1), 2C (Thy-1), and 2D (cadherin) were shown in the Supplemental Fig. S2. *p < 0.05. Con, control; GÖ, Gö6976; RÖ, Röttlerin; Sc siRNA, scramble small interference RNA.

Phorbol esters are known to bind directly to PKC protein by mimicking the natural ligand diacylglycerol, and PKC protein has been reported to be involved in the induction of Thy-1^16–18^. We further investigated which isoform of PKC protein is involved in the regulation of Thy-1 expression in vascular endothelial cells. Pre-treatment with Röttlerin (5 μM), a PKC-δ inhibitor, abolished the PMA-induced PKC-δ activation (Fig. 2A) and Thy-1 up-regulation (Fig. 2B) in HUVECs. However, pre-treatment of HUVEC with 200 nM of Gö6976, a PKC-α/β inhibitor, did not significantly affect the PMA-induced Thy-1 up-regulation (Fig. 2B), suggesting that the PKC-δ isoform may participate in the regulation of Thy-1 expression. We also used the PKC-δ siRNA to confirm that PMA increased Thy-1 expression through a PKC-δ-mediated pathway. As shown in Fig. 2C, transfection with PKC-δ siRNA significantly reduced the PKC-δ expression and PMA-induced Thy-1 up-regulation. The Röttlerin-prevented PMA-induced Thy-1 up-regulation was also observed in HPMECs (data not shown). Figure 2D shows that PMA (20 ng/mL) treatment increased membrane translocation of PKC-δ (top panel), but did not significantly affect the level of nuclear PKC-δ (bottom panel) in HUVECs. To further confirm the involvement of PKC-δ in regulating the Thy-1 gene expression, luciferase activity assay was conducted. As illustrated in Fig. 2E, pre-treatment of HUVECs with Röttlerin abolished the PMA-increased Thy-1 promoter activity. Wound-healing assay also demonstrated that Röttlerin, but not Gö6976, abolished the PMA-induced migration inhibition in HUVECs (Fig. 2F). Since tube formation is also a critical step of angiogenesis, we further examined the effect of Röttlerin and Gö6976 on the capillary-like tube formation. As shown in Fig. 2G, PMA (20 ng/mL) interrupted the capillary-like tube formation, and this effect was abolished by pre-treatment with Röttlerin, but not Gö6976, suggesting that PKC-δ is also involved in PMA-induced interruption of capillary-like tube formation.

### Involvement of Syk activation in the PMA-induced Thy-1 up-regulation and migration inhibition in endothelial cells

**Figure 3:**
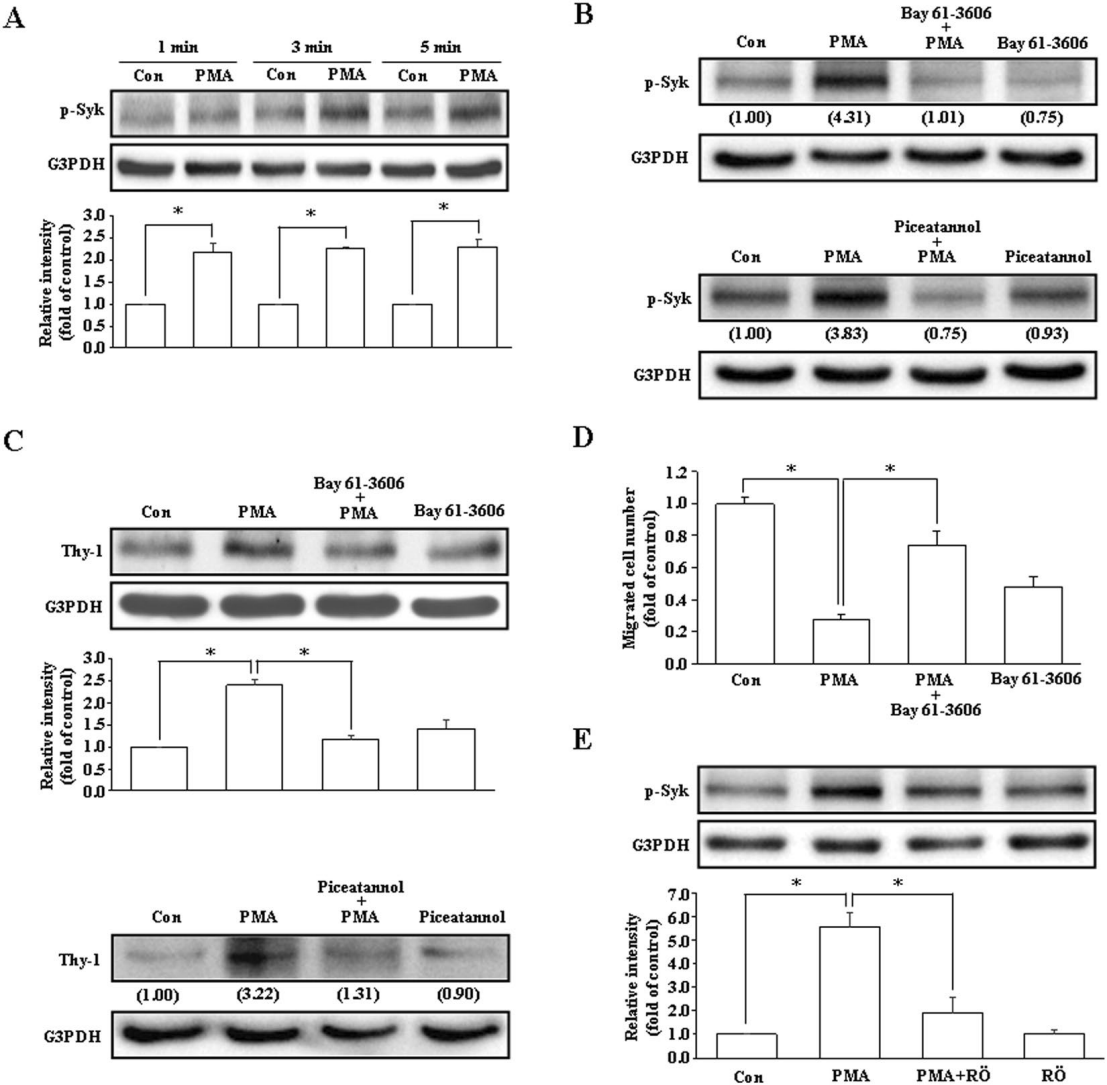
Activation of Syk is required for PMA-induced Thy-1 up-regulation. (**A**) PMA (20 ng/mL) time-dependently increased Syk phosphorylation in HUVECs. n = 3. Pre-treatment of HUVECs with Syk inhibitors, Bay 61-3606 (1 μM) or piceatannol (10 μM), abolished the PMA-induced Syk activation (**B**), Thy-1 up-regulation (**C**) and migration inhibition (**D**). In (**B**), values shown in parentheses represent the quantified results adjusted with G3PDH and expressed as ratio over control. In (**C** and **D**), n = 3. (**E**) Pre-treatment of HUVECs with Röttlerin (5 μM) abolished the PMA-induced increases in the levels of p-Syk protein. n = 3. The gels have been run in the same experimental conditions and the cropped blots were shown. The entire gel pictures of 3A (p-Syk), 3B (p-Syk), 3C (Thy-1), and 3E (p-Syk) were shown in the Supplemental Fig. S3. *p < 0.05. Con, control; RÖ, Röttlerin.

Since Syk-mediated signals have been shown to be the downstream of PKC-δ-mediated thrombin-induced intercellular adhesion molecule-1 (ICAM-1) expression in endothelial cells^19^, we examined whether activation of Syk is involved in the PMA-induced Thy-1 up-regulation. As shown in Fig. 3A, treatment with PMA (20 ng/mL) for 1–5 min increased phosphorylation of Syk at Tyr525/526 in HUVECs. Pre-treatment with Syk inhibitors, Bay 61-3606 (1 μM) or piceatannol (10 μM), abolished the PMA-induced Syk activation (Fig. 3B) and Thy-1 up-regulation (Fig. 3C) in HUVECs, suggesting the involvement of Syk activation in the PMA-induced up-regulation of Thy-1. We conducted a wound-healing assay to verify whether Syk is involved in the PMA-induced migration inhibition in endothelial cells. As illustrated in Fig. 3D, pre-treatment with Bay 61–3606 (1 μM) prevented the PMA-induced migration inhibition in HUVECs. To examine whether Syk is a downstream molecule of PKC-δ, HUVECs were pre-treated with the PKC-δ inhibitor followed by PMA treatment. As demonstrated in Fig. 3E, pre-treatment with Röttlerin prevented PMA-induced increases of Syk phosphorylation in HUVECs, suggesting that Syk is a downstream molecule of PKC-δ.

### Involvement of NF-κB (p65) activation in the PMA-induced up-regulation of Thy-1 in HUVECs

**Figure 4:**
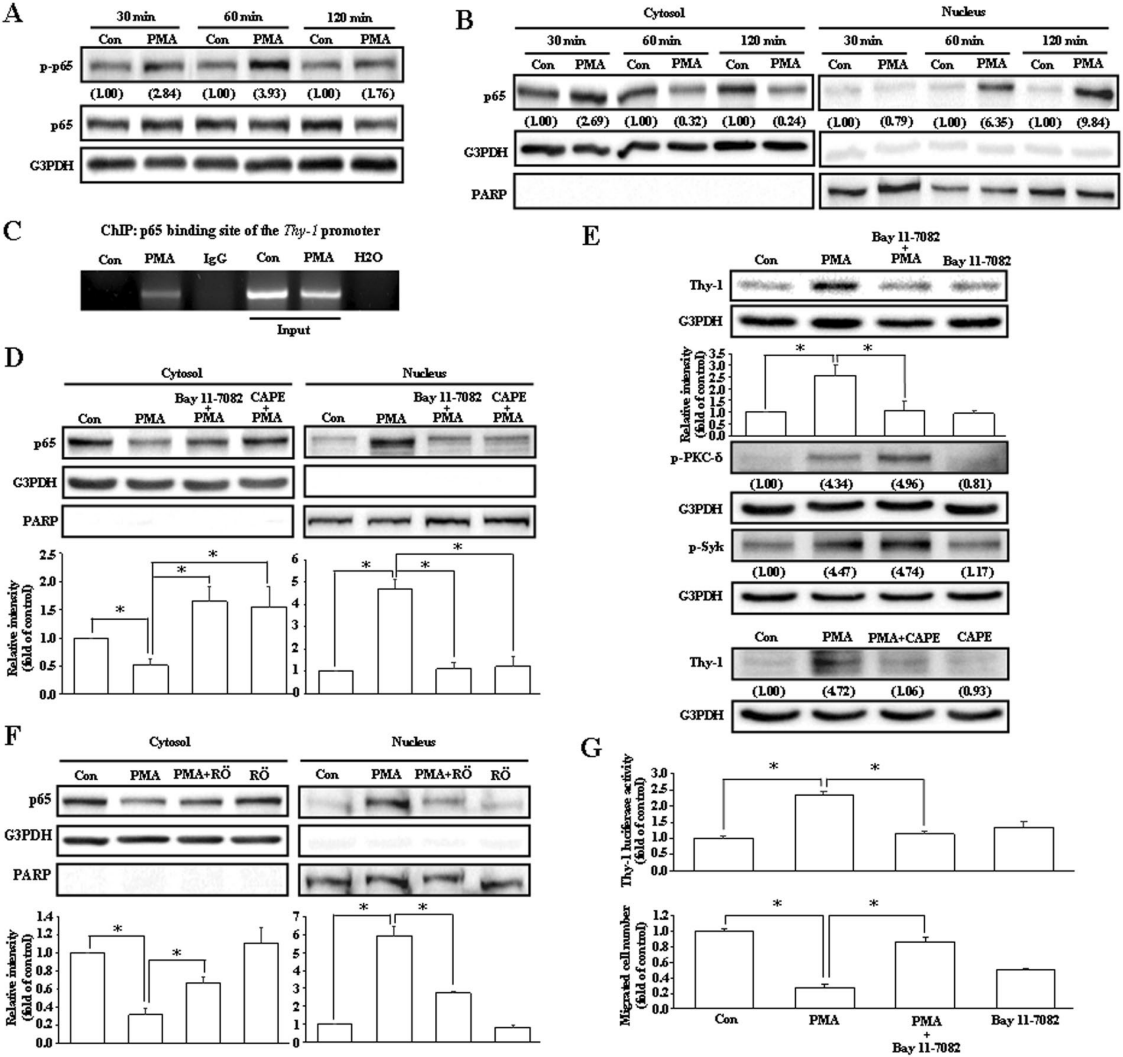
Involvement of NF-κB (p65) in the PMA-induced Thy-1 up-regulation. (**A**) PMA (20 ng/mL) time-dependently increased the levels of p-p65 protein in HUVECs. Values shown in parentheses represent the quantified results adjusted with G3PDH and expressed as ratio over control. (**B**) Treatment with PMA for 30–120 minutes increased nuclear translocation of p65. PARP and G3PDH were used as a nuclear and cytosolic protein marker, respectively, to confirm the purities of isolation. Values shown in parentheses represent the quantified results adjusted with G3PDH or PARP and expressed as ratio over control. (**C**) PMA treatment increased the binding of p65 onto the Thy-1 promoter in HUVECs. (**D**) Pre-treatment with NF-κB inhibitors, Bay 11-7082 (10 nM) or CAPE (10 μM), prevented the PMA-induced p65 nuclear translocation. The quantified results were adjusted with G3PDH or PARP and expressed as ratio over control. n = 3. (**E**) Pre-treatment with Bay 11-7082 (top panel) or CAPE (bottom panel) abolished the PMA-induced up-regulation of Thy-1. However, the PMA-induced activations of PKC-δ and Syk were not significantly affected by Bay 11-7082 treatment. In top panel, n = 3. In bottom panel, values shown in parentheses represent the quantified results adjusted with G3PDH and expressed as ratio over control. (**F**) Pre-treatment with Röttlerin (5 μM) significantly reduced the PMA-induced p65 nuclear translocation in HUVECs. n = 3. (**G**) Pre-treatment with Bay 11-7082 prevented the PMA-induced increases of the Thy-1 promoter activity (top panel) and migration inhibition (bottom panel) in HUVECs. The luciferase activity was normalized by Renilla luciferase expression. The quantified results were expressed by fold of control. n = 4 for luciferase activity assay and n = 3 for migration assay. The gels have been run in the same experimental conditions and the cropped blots were shown. The entire gel pictures of 4A (p-p65) and 4E (Thy-1 and p-PKC-δ) were shown in the Supplemental Fig. S4. *p < 0.05, different from corresponding control. ^#^*p* < 0.05, different from PMA-treated. CAPE, Caffeic acid phenethyl ester; Con, control; RÖ, Röttlerin.

To determine which transcription factor is involved in the PMA-increased Thy-1 expression, the sequence of Thy-1 promoter region was analyzed using promoter prediction database. The results show that NF-κB (p65)-binding sites were found on the Thy-1 promoter region. Initially, we examined whether PMA treatment can induce activation of p65, which is the DNA binding subunit of the NF-κB transcription factor protein complex. As demonstrated in Fig. 4A, PMA increased phosphorylation of p65 at Ser529 in HUVECs. We further investigated whether p65 is involved in the PMA-induced Thy-1 up-regulation. Since activated p65 can translocate from the cytosol to the nucleus where p65 binds to the promoter and functions as a transcriptional activator, we examined whether PMA can induce p65 nuclear translocation. As illustrated in Fig. 4B, PMA increased translocation of p65 from the cytosol to the nucleus in a time-dependent manner. To examine whether p65 protein can directly regulate the Thy-1 promoter activity, the chromatin immunoprecipitation (ChIP) assay was conducted. As shown in Fig. 4C, PMA increased the binding of the p65 protein onto the Thy-1 promoter. Pre-treatment of HUVECs with NF-κB inhibitors, Bay 11-7082 or caffeic acid phenethyl ester (CAPE), abolished the PMA-induced p65 nuclear translocation (Fig. 4D) and Thy-1 up-regulation (Fig. 4E). The Bay 11-7082-prevented PMA-induced Thy-1 up-regulation was also observed in HDMECs (data not shown). However, pre-treatment with Bay 11-7082 did not affect the PMA-induced activations of PKC-δ and Syk in HUVECs. On the other hand, pre-treatment of HUVECs with Röttlerin inhibited PMA-induced p65 nuclear translocation (Fig. 4F). These data suggest that PKC-δ/Syk are the upstream regulators of p65. Figure 4G demonstrates that pre-treatment of HUVECs with Bay 11-7082 (10 nM) also abolished the PMA-induced increases of Thy-1 promoter activity (upper panel) and migration inhibition (bottom panel).

### Effects of PMA on angiogenesis of zebrafish embryos

**Figure 5:**
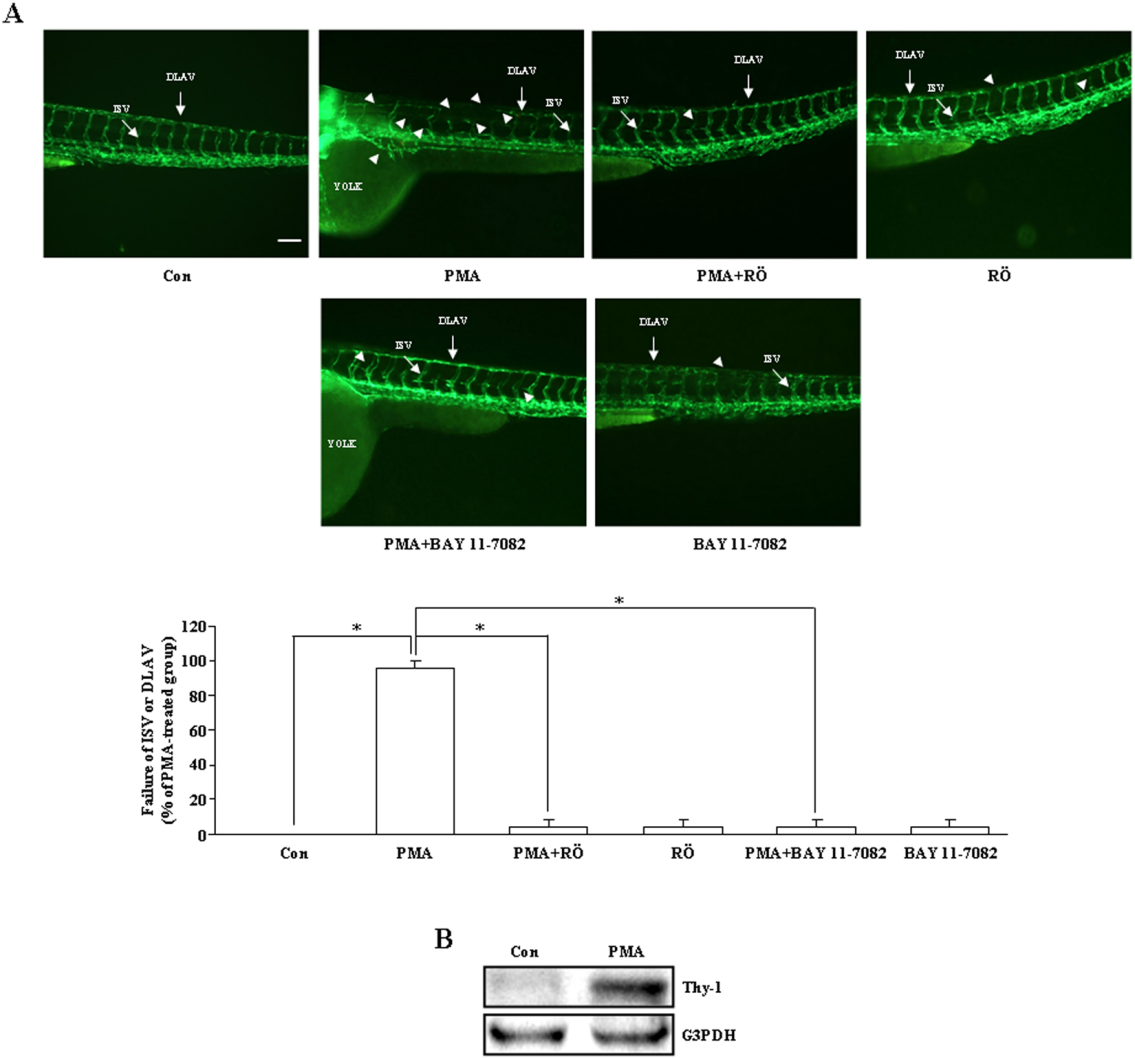
Effects of Röttlerin and Bay 11-7082 on the PMA-inhibited angiogenesis. (**A**) Treatment with PMA at 72 hours after post-fertilization significantly inhibited angiogenesis, and this inhibition was suppressed by co-treatment with Röttlerin (5 μM) or Bay 11-7082 (10 nM). Embryos were examined at 3 days post-fertilization. More than 20 embryos were examined in each experimental group. Twenty dechorionated Tg(fli1:EGFP)^y1^ embryos were grown in a 6-well plate, and incubated in 2 mL solution containing PMA (20 ng/mL). Top panel: fluorescent images show defective vasculatures in ISV and DLAV at the PMA-treated Tg(fli1:EGFP)^y1^ embryos. The arrowheads indicate the failure of forming the ISV or DLAV in the PMA-treated Tg(fli1:EGFP)^y1^ embryos. Bar = 100 μm. Bottom panel: quantified results expressed by percentage of total ISV. (**B**) The expression of Thy-1 was detected in zebrafish embryos at 72 hours post-fertilization by Western blot analysis. The cropped blot was shown. The entire gel picture of 5B (Thy-1) was shown in the Supplemental Fig. S5. RÖ, Röttlerin.

We used Tg(fli1:EGFP)^y1^ zebrafish embryos to examine the effect of PMA on angiogenesis. As shown in Fig. 5A, the failure in forming the intersegmental vessels (ISV) and the dorsal longitudinal anastomotic vessels (DLAV) was observed in the PMA-treated embryos. However, co-treatment of zebrafish embryos with PMA and Röttlerin or Bay 11-7082 significantly reduced the PMA-induced interruption of angiogenesis. Western blot analysis was performed to verify the effect of PMA on Thy-1 expression in zebrafish embryos. As shown in Fig. 5B, Thy-1 protein was detected in the PMA-treated zebrafish embryos, but not un-treated control embryos.

### Effects of short-term PMA treatment on endothelial cell migration

**Figure 6:**
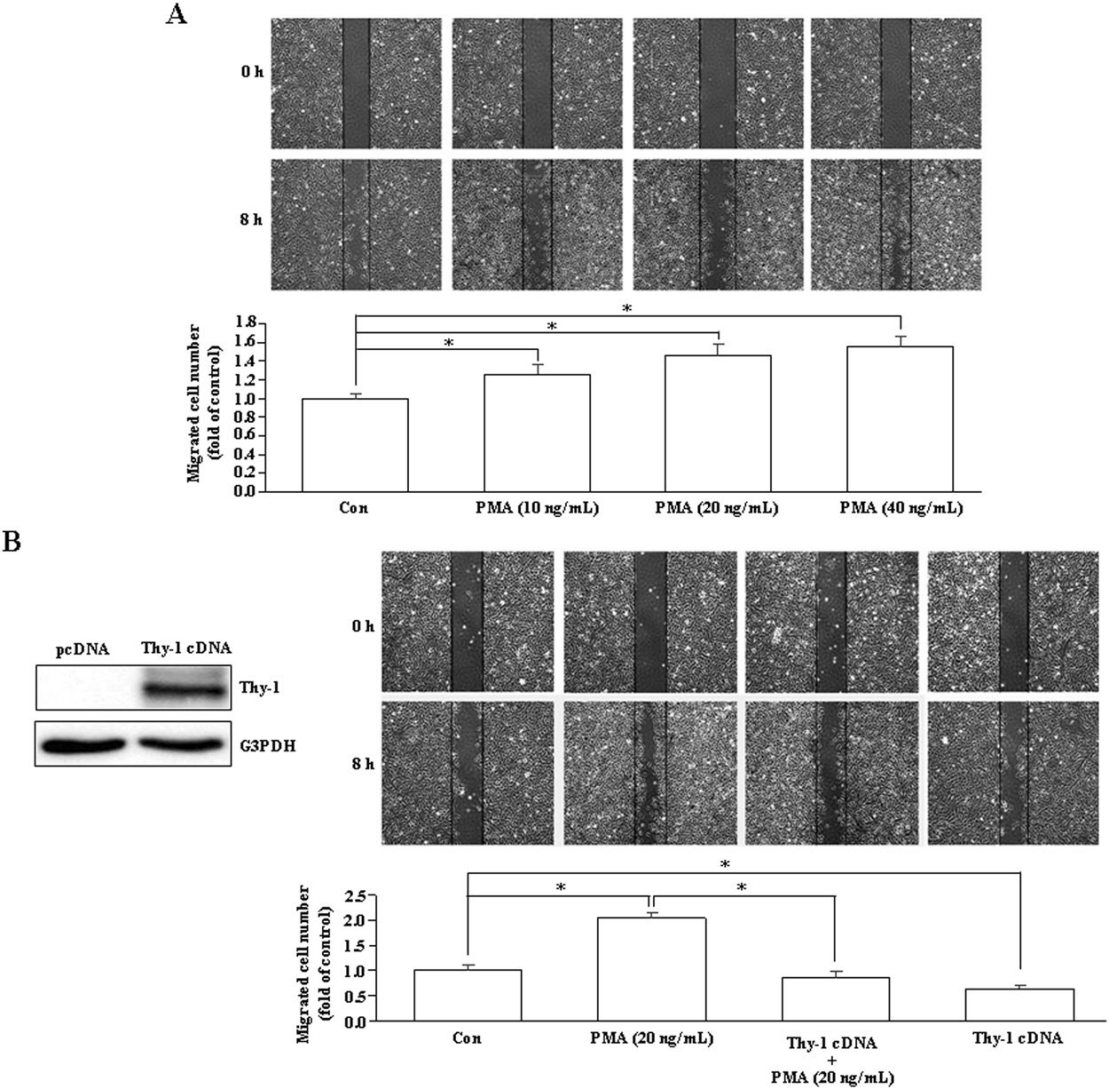
Short-term PMA treatment enhances endothelial cell migration. (**A**) Treatment with PMA (10–40 ng/mL) for 8 hours, concentration-dependently enhanced endothelial cell migration. n = 7. (**B**) The PMA-enhanced migration was not observed in the Thy-1-overexpressed HUVECs. n = 4. *p < 0.05. Left panel showed overexpression of Thy-1 protein in HUVECs transfected with Thy-1 cDNA. The cropped blot was shown. The entire gel picture of 6B (Thy-1) was shown in the Supplemental Fig. S6. Con, control.

In the present study, our data demonstrated that PMA inhibited migration of endothelial cells *in vitro* and induced anti-angiogenesis *in vivo*. However, PMA was reported to be able to induce angiogenesis^20^. In our studies described above, endothelial cells were treated with PMA for 24 hours to induce Thy-1 up-regulation followed by additional 8–10 hours PMA treatment during wound healing migration assay. Since phorbol esters-induced Thy-1 up-regulation in endothelial cells was not observed until 20–28 hours after treatment^18^, we tested whether induction of Thy-1 expression contributed to the PMA-induced anti-angiogenesis by examining the migration activity of HUVECs after treatment with PMA for 8–10 hours when the Thy-1 expression was not up-regulated. As shown in Fig. 6A, treatment with PMA (10–40 ng/mL) for 8–10 hours, concentration-dependently enhanced endothelial cell migration. However, the PMA-enhanced endothelial cell migration was not observed in Thy-1-overexpressed endothelial cells treated with PMA (Fig. 6B), suggesting that PMA inhibited endothelial cell migration through up-regulation of Thy-1.

## Discussion

We previously demonstrated that Thy-1 can serve as a marker for adult angiogenesis^13^. We also showed that expression of Thy-1 can inhibit endothelial cell migration, but did not affect endothelial cell proliferation^14^. In the present study, we used PMA as a Thy-1 inducer to further investigate how the Thy-1 expression is regulated. Our data show that PMA upregulated Thy-1 expression through activating the PKC-δ/Syk/NF-κB pathway in endothelial cells. To our knowledge, this is first demonstration that the PKC-δ/Syk/NF-κB pathway is involved in regulating Thy-1 expression in endothelial cells.

PMA was shown to up-regulate Thy-1 expression in HDMECs^15^. In this study, we demonstrated that PMA (20 ng/mL) treatment increased the levels of Thy-1 mRNA (Fig. 1A) and protein (Fig. 1B) and the promoter activity (Fig. 1C) in various human vascular endothelial cells, suggesting that PMA may increase Thy-1 expression at the transcriptional level. We previously showed that PMA can reduce endothelial cell migration, and this reduction was abolished by Thy-1 siRNA^14^. In this study, we further demonstrated that short-term PMA treatment can enhance endothelial cell migration and overexpression of Thy-1 prevented migration enhancement induced by short-term PMA treatment. Taken together, these findings suggest that PMA caused migration inhibition in endothelial cells through up-regulation of Thy-1 expression.

It has been shown that PMA can bind directly to PKC protein by mimicking the natural ligand diacylglycerol, and phosphorylate PKC protein in HUVECs^20^. PKC is a family of protein kinase and has been reported to be involved in regulation of the angiogenic response^20,21^. In the present study, we demonstrated that blockade of the PKC-δ activity, but not PKC-α/β activity, abolished the PMA-induced increases of Thy-1 protein level (Fig. 2B) and promoter activity (Fig. 2E), interruption of the capillary-like tube formation (Fig. 2G), and migration inhibition (Fig. 2F) in HUVECs, suggesting that activation of PKC-δ, but not PKC-α/β, participated in the PMA-induced Thy-1 up-regulation.

In the present study, our data suggest that Syk is the downstream molecule of PKC-δ in the PMA-induced up-regulation of Thy-1 expression. Syk, a non-receptor tyrosine kinase, has been demonstrated to play a crucial role in innate and adaptive immunity^22^ and to participate in the integrin signaling which is associated with cell adhesion and migration^23^. Previous studies have shown that PKC can activate AMP-activated protein kinase (AMPK), hence leading to activation of Syk in the PMA-mediated adhesion of monocytes^24^. PKC has the ability to phosphorylate Syk and enhance its tyrosine kinase activity^25^. Moreover, PKC-δ can activate Syk in thrombin-induced ICAM-1 expression in endothelial cells^19^. In the present study, we demonstrated that pre-treatment with Syk inhibitors abolished the PMA-induced Thy-1 up-regulation (Fig. 3C) and migration inhibition (Fig. 3D), suggesting that Syk is involved in the regulation of PMA-induced Thy-1 up-regulation. Moreover, pre-treatment of HUVECs with Röttlerin (5 μM), a PKC-δ inhibitor, abolished the PMA-induced Syk phosphorylation (Fig. 3E), suggesting that Syk is a downstream molecule of PKC-δ.

Transcription factors are crucial proteins for regulating gene expression. Using promoter prediction database for Thy-1 promoter sequence analysis and by comparing the consensus sequence of NF-κB on the Thy-1 promoter sequence, we found that there are four putative NF-κB (p65)-binding sites on the Thy-1 promoter region^26,27^. Pre-treatment with p65 inhibitors abolished the PMA-increased Thy-1 protein level (Fig. 4E) and promoter activity (Fig. 4G) in vascular endothelial cells, suggesting that p65 might play an essential role in regulating Thy-1 expression. This notion was supported by the evidence that PMA increased recruitment of p65 to the Thy-1 promoter region demonstrated by ChIP assay (Fig. 4C). In unstimulated cells, the p65 was retained in the cytoplasm. After stimulation, p65 translocates from the cytosol to the nucleus, and then binds onto the gene promoter to regulate gene expression^28^. Besides, p65 phosphorylation has been reported to play a crucial role in controlling the p65 transactivation activity^29^. In the present study, we showed that PMA induced phosphorylation (Fig. 4A) and nuclear translocation of p65 (Fig. 4B) in HUVECs. Pre-treatment with Bay 11-7082 prevented the PMA-induced migration inhibition (Fig. 4G). These data suggest that activation of NF-κB contributed the PMA-induced increases of Thy-1, which in turn caused migration inhibition in vascular endothelial cells.

Previous studies have shown that PKC-δ is predominantly in the cytoplasm of non-apoptotic cells; nuclear translocation of PKC-δ mediates caspase-3 upregulation and is essential to induce apoptosis^30–33^. Moreover, PKC-δ has been reported to interact with p65, and then translocated into the nucleus to up-regulate some pro-inflammatory chemokines^34^. However, we found that PMA treatment increased membrane translocation of PKC-δ, but did not significantly affect the level of nuclear PKC-δ (Fig. 2D). Moreover, Röttlerin, a PKC-δ inhibitor, abolished the PMA-induced p65 nuclear translocation (Fig. 4F), and Bay 11-7082, a NF-κB inhibitor, did not affect the PMA-induced activations of PKC-δ and Syk (Fig. 4E). These data suggest that PKC-δ/Syk are the upstream regulators of p65. Our findings are similar to a previous study demonstrating that PKC-δ/Syk/p65 signaling pathway regulates the thrombin-induced intercellular adhesion molecule-1 (ICAM-1) expression in endothelial cells^19^.

We previously showed that endothelial Thy-1 expression is up-regulated by cytokines, such as tumor necrosis factor-α and interleukin-1β (IL-1β), but not growth factors, such as vascular endothelial growth factor (VEGF) and basic fibroblast growth factor (bFGF)^13,18^. In our previous study, we also demonstrated that IL-1β can reduce endothelial cell migration, and this reduction was abolished by transfection of the cell with Thy-1 siRNA^14^, suggesting that the inhibitory effect of Thy-1 in angiogenesis is not only limited to PMA treatment. In the present study, we also examined whether expression of Thy-1 could affect embryonic vasculature development. Using the zebrafish model, we discovered that the Thy-1 protein level was un-detectable in the absence of PMA treatment, but significantly increased in the presence of PMA (Fig. 5B). PMA treatment also interrupted the vascular development in the zebrafish embryo, and the PMA-induced anti-angiogenesis was abolished by co-treatment with Röttlerin, a PKC-δ inhibitor, or Bay 11-7082, a p65 inhibitor (Fig. 5A). These results are consistent with the *in vitro* study, suggesting that Thy-1 may have the ability to terminate the angiogenic processes and overexpression of Thy-1 might be translated into a potential therapy to treat diseases involving pathologic angiogenesis.

The effect of Thy-1 in cellular adhesion of leukocytes and tumor cells to endothelial cells has attracted the attention of researchers. Thy-1 has been reported to be a regulator of cell-cell and cell-matrix interaction^9^. It has been shown that Thy-1-expressed vascular endothelial cells interact with αvβ3 integrins on melanoma cells, and then promote melanoma cells migration through an endothelial cell monolayer^35^. Thy-1-expressed lymphatic endothelial cells mediate cell adhesion of leukocyte to lymphatic endothelium^36^. Moreover, the interaction between Thy-1 and leukocyte integrin Mac-1 mediates the transendothelial cell migration of leukocyte^37^. On the contrary, Thy-1 has been shown to inhibit fibroblasts adhesion and migration by modulating p190 RhoGAP and RhoGTPases^38^. These findings suggest that Thy-1 might have diverse roles in regulating cell adhesion. In our previous study, we demonstrated that overexpression of Thy-1 reduced adhesion and migration of endothelial cells on collagen-coated plates^14^, suggesting that Thy-1 plays a negative-regulated role in endothelial cell migration. The role of Thy-1 in cell-cell interaction and the mechanism of Thy-1-integrin interaction during angiogenesis deserves further investigation.

PMA, the most commonly used phorbol ester, has been shown to be a strong inducer of sprouting angiogenesis^39,40^. Previously, Osaki, *et al*. demonstrated that PMA induces HUVECs to form luminal structures^41^. In contrast to their study, our results showed that PMA interrupted capillary-like tube formation (Fig. 2G). The discrepancy might be due to the experimental design. In their study, HUVECs were seeded on a collagen gel and exposed to PMA in the culture medium. However, in our study, HUVECs were treated with PMA for 24 hours to induce the Thy-1 expression, and then seeded onto a layer of Matrigel. The protein level of Thy-1 was increased by 24 hours treatment with PMA. Consequently, the interruption of the capillary-like tube formation might due to the function of Thy-1.

Previously, Shih *et al*. showed that PMA induces PCK-α- and PCK-ζ-dependent up-regulation of VEGF mRNA in human glioblastoma cells via a post-transcriptional mRNA stabilization mechanism, but not transcriptional activation of VEGF^42^. The induced up-regulation of VEGF mRNA begins at 1 hour and peaked at 8 hours after PMA treatment. Consistent with their findings, our data showed that treatment with PMA (20 ng/mL) for 8–10 hours enhanced endothelial cell migration (Fig. 6A). However, pre-treatment with PMA (20 ng/mL) for 24 hours to allow the up-regulation of Thy-1 prior to wound healing assay followed by additional 8–10 hours PMA (20 ng/mL) treatment to allow cell migration to close the wound caused migration inhibition, suggesting that a longer treatment of PMA to induce migration inhibition was due to Thy-1 up-regulation. This notion was supported by the evidence showing that a short term of PMA treatment did not enhance the migration in the Thy-1-overexpressed HUVECs (Fig. 6B). Moreover, we previously showed that the PMA-induced migration inhibition was abolished by transfection of HUVECs with Thy-1 siRNA^14^. Our previous study demonstrated that VEGF did not significantly affect the level of Thy-1 mRNA in vascular endothelial cells^13^. In the present study, we demonstrated that treatment with PMA (20 ng/mL) for 20–28 hours increased the levels of Thy-1 protein through activation of PKC-δ. These findings led us to hypothesize that PMA might increase VEGF secretion through activation of PKC-α and PKC-ζ to induce angiogenesis, and then induced Thy-1 expression through activation of PKC-δ to terminate the angiogenesis processes. This concept is similar to vasohibin-1 (VASH1) as a negative feedback regulator of angiogenesis. Previously, Sato demonstrated that VEGF- and FGF-2-induced vasohibin-1 (VASH1) is a negative feedback regulator of angiogenesis^43^. The angiogenic factors-induced VASH1 is mainly expressed in the endothelial cells at the site of angiogenesis. Moreover, VEGF- and FGF-2-induced VASH1 has been reported to inhibit the cell migration, proliferation, and tube formation *in vitro* and angiogenesis *in vivo*^44^. The VASH1-expressed endothelial cells terminate the angiogenesis at the termination zone of angiogenesis^45^. The findings of the present study suggest that Thy-1 might also play a role in termination of angiogenesis.

We previously showed that Thy-1 is expressed on vascular endothelial cells in settings of pathological angiogenesis in adult animals and a specific setting of physiological angiogenesis, but not during embryonic angiogenesis^13^. We also demonstrated that Thy-1 is upregulated in cultured vascular endothelial cells treated with inflammatory cytokines, but not growth factors. Thy-1 is a N-glycosylated glycophosphatidylinositol anchored cell surface protein. Although Thy-1 lacks a transmembrane region, several studies have suggested that Thy-1 might participate in tyrosine kinase signaling pathway^9,46^. Whether Thy-1 directly interacts with cytoplasmic tyrosine kinases or indirectly interacts with signaling molecules are still unclear. Integrins have been reported to play an important role in Thy-1 signaling. The interaction between Thy-1 and αVβ3 integrin mediated the communication between neurons and astrocyte, which in turn stimulated astrocyte migration^47^. However, the Thy-1 signaling and the function of Thy-1/integrin interaction in vascular endothelial cell migration and angiogenesis still need further investigation. Although studies of molecular mechanisms underlying Thy-1-inhibited endothelial cell migration and tube formation are still ongoing, the results from our previous study showed that Thy-1 inhibited migration and capillary-like tube formation in vascular endothelial cells through reducing the RhoA activity^14^.

In conclusion, the results of our study suggest that PMA might have biphasic effects on angiogenesis; PMA initially enhanced endothelial cell migration, subsequently activating the PKC-δ/Syk/NF-κB pathway to up-regulate Thy-1 expression, which in turn caused endothelial cell migration inhibition. Understanding the regulation of Thy-1 expression and the function of Thy-1 may lead to useful therapies for angiogenic disorders.

## Methods

### Chemicals

Anti-p-p65 antibody, Gö6976, heparin, PMA, Röttlerin, and PKC-δ siRNA were purchased from Sigma-Aldrich (St.Louis, MO). Bay 11-7082, Bay 61-3606, piceatannol, and caffeic acid phenethyl ester (CAPE) were purchased from Cayman Chemicals (Ann Arbor, MI). Lipofectamine 3000 reagent was acquired from Invitrogen Life Technologies (Gaithersburg, MD). Antibody specific for Thy-1, and p-PKC-δ was purchased from Cell Signaling Technology (Danvers, MA). Anti-Syk, p65, PKC-δ, and G3PDH were purchased from Santa Cruz Biotechnology (Santa Cruz, CA). Antibody specific for p-Syk was purchased from EMD Millipore (Temecula, CA). Dual-Luciferase assay kit was purchased from Promega (Madison, WI). Penicillin and streptomycin were purchased from HyClone (South Logan, Utah). Endothelium cell growth factor (ECGS) was purchased from Biomedical Technologies (Stoughton, MA). Fetal calf serum (FCS), Medium 199 (M199), and GlutaMAX Supplement were obtained from GIBCO (Grand Island, NY). Endothelial Cell Growth Medium MV was purchased from PromoCell (Heidelberg, Germany).

### Cell culture

Human umbilical vein endothelial cells (HUVECs) purchased from Bioresource Collection and Research Center (BCRC) in Taiwan were grown in M199, supplemented with 10**%** FCS, ECGS (30 mg/L), heparin (5 units/L), HEPES (10 mmol/L), penicillin (100 U/mL), and streptomycin (100 μg/ mL). Cells from passages 3–10 were used. HDMECs were grown in Endothelial Cell Growth Medium MV supplemented with 5**%** FCS, ECGS, EGF (10 ng/mL), heparin (90 μg/mL), hydrocortisone (1 μg/mL), penicillin (100 U/mL), and streptomycin (100 μg/mL). HPMECs was kindly provided by Dr. C. James Kirkpatrick (Johannes-Gutenberg University, Mainz, Germany)^48,49^ and grown in M199 containing 20**%** FCS, ECGS (30 mg/L), heparin (5 units/L), HEPES (10 mmol/L), GlutaMAX Supplement (2 mM), penicillin (100 U/mL), and streptomycin (100 μg/ mL).

### Preparation of Thy-1 promoter plasmid construct

The fragment of human Thy-1 promoter region spanning positions −651 to +424 was amplified by PCR using HUVEC genomic DNA as a template. The following primers were used: forward 5′-TAGGTACCACTCAGTCCTTTTTGTGCTGTC-3′ and reverse 5′-CCAAGCTTACTGGACTGGGGTCTTCCGCTG-3′. Thy-1 promoter was subcloned into the pGL3-Basic luciferase reporter vector at HindIII/Kpn1 sites.

### Cell transfection

For transient transfection of Thy-1 promoter constructs or Thy-1 pcDNA 3.1 into human vascular endothelial cells, Lipofectamine 3000 was used according to the manufacturer’s protocol. Briefly, Lipofectamine 3000/DNA mixture was incubated at room temperature for 15 minutes. The mixture was then added dropwise to the DMEM-GlutaMAX Supplement medium containing 2**%** FCS and 0.02**%** ECGS, and incubated in a humidified 37 °C incubator for 6 hours. The culture medium was replaced, and the cells were incubated for additional 24 hours. For transient transfection of PKC-δ siRNA or scramble siRNA (control) into HUVECs, Lipofectamine 3000 was used according to the manufacturer’s protocol. Briefly, Lipofectamine 3000/siRNA mixture was incubated at room temperature for 10 minutes, and then added dropwise to the M199 culture medium supplemented with 10% FCS and ECGS (30 mg/L), and incubated in a humidified 37 °C incubator for 40 hours.

### Thy-1 luciferase assay

After transfection with Thy-1 promoter plasmid (0.5 μg) and Renilla plasmid (50 ng), the cells were treated with PMA (20 ng/mL), washed with ice-cold phosphate-buffered saline (PBS), and then lysed in passive lysis buffer. Luciferase activity was measured using the Dual-Luciferase Reporter Assay kit and recorded by 20/20n luminometer (Turner Biosystems, Sunnyvale, CA)

### Western blot analysis

Protein extraction and Western blot analysis were performed as previously described^50^. The intensity of each band was quantified by densitometry analysis using Image Pro Plus 4.5 software. In each figure, the gels have been run under the same experimental conditions and cropped blots were shown. The methods were carried out in accordance with the approved guidelines.

### RNA isolation and RT-PCR

The total RNAs were isolated using Cell Total RNA Mini Kit (Geneaid, TW) according to the manufacturer’s protocol. Total RNAs (1 μg) were reversely transcribed to synthesize cDNA using iScrip cDNA Synthesis Kit (Bio-Rad, CA). After that, 2 μL of cDNA was used for PCR reaction with specific primers for Thy-1. The following primers were used: forward: CAGCAGTTCACCCATCCAGT; reverse: ACCAGTTTGTCTCTGAGCACT. The PCR products were electrophoresed on 1.5**%** agarose gel.

### Wound healing migration assay

Wound healing migration assay was performed as previously described with minor modification^51^. Briefly, HUVECs (1.5 × 10^4^) were seeded in Culture-Insert (Martinsried, Germany) coated with 1**%** gelatin and placed in 3.5 cm culture dish. After cell attachment and chemical treatment, the Culture-Insert was gently removed. The culture dish was washed with PBS to remove non-adherent cells or cells debris, and then filled with M199 culture medium containing the endothelium cell growth factor supplement at 37 °C incubator. The cells were allowed to migrate for 8–10 hours, and then examined their migration activity under a light microscope.

### Cytoplasmic and nuclear extract preparation

Cells were washed with ice-cold PBS buffer twice, and then scraped with PBS. After centrifugation at 6,500 rpm for 10 minutes, the supernatants were discarded. The cytoplasmic and nuclear proteins were extracted using cytoplasmic and nuclear protein extraction kit (BIOTOOLS CO., LTD. Taiwan) following manufacturer’s protocol.

### Chromatin immunoprecipitation assay (ChIP assay)

The ChIP assay was performed as previously described^52^. Primers for consensus p65 binding site in the Thy-1 promoter were: forward: TGGGCTCCAGCTGAAAAGGA; reverse: GCCAATCAGAGGCGGAGATT. All the PCR products were analyzed on 1.5**%** agarose gels.

### Capillary-like tube formation assay

Capillary-like tube formation assay was performed as previously described^14^. Briefly, the wells of ibidi μ–Slide Angiogenesis (Martinsried, Germany) were coated with 10 μL of Matrigel (10 mg/mL) (BD Bioscience Pharmigen, CA, USA) and incubated at 37 °C for 30 minutes. HUVECs (6 × 10^3^/well) were suspended in M199 culture medium containing the endothelium cell growth factor supplement and plated onto a layer of Matrigel. The ibidi μ–Slide Angiogenesis was then incubated for an additonal 4 hours at 37  C, and capillary-like tube formation was observed with a microscope.

### Zebrafish animal model

Transgenic Tg(fli1:EGFP)^y1^ embryos of zebrafish (*Danio rerio*) obtained from the zebrafish core facility of Taipei Medical University were maintained at 28 °C on a 14 hours light/10 hours dark cycle, and used to track the effects of PMA on the development of angiogenesis^53^. The developmental stages of the embryos were determined according to The Zebrafish Book^54^. The experimental procedures in studying zebrafish were approved by Taipei Medical University Institutional Animal Care and Utilization Committee (TMU-IACUC) and all experiments were performed in accordance with relevant guidelines and regulations. The zebrafish embryos were treated with 2 mL of PMA (20 ng/mL), or PMA combined with Röttlerin (5 μM), a PKC-δ inhibitor, or Bay 11-7082 (10 nM), a p65 inhibitor, at 20 hpf (hours post-fertilization) for 72 hours. The effects of these chemicals on the development of angiogenesis in zebrafish embryos were examined under an inverted fluorescence microscope.

### Statistical analysis

All data were presented as the mean value ± s.e.mean from three to four independent experiments. Comparisons were subjected to one-way analysis of variance (ANOVA) followed by Fisher’s least significant difference test. *P* < 0.05 was considered statistically significant.

## Electronic supplementary material

Supplemental Figures

## Data Availability

All the data generated and/or analysed during the current study are included in this article and the Supplementary Information file, and are available from the corresponding author on reasonable request.
