# Supplementary material for: PMA inhibits endothelial cell migration through activating the PKC-δ/Syk/NF-κB-mediated up-regulation of Thy-1
Source: Sci Rep. 2018 Nov 2;8:16247. doi: 10.1038/s41598-018-34548-8 (PMC6214930; doi:10.1038/s41598-018-34548-8)
Supplement: Supplementary file 1 — Supplemental Figures [file 41598_2018_34548_MOESM1_ESM.pdf]

PMA inhibits endothelial cell migration through activating the PKC-δ/Syk/NF-κB-mediated up-regulation of Thy-1

Heng-Ching Wen[1], Yen Nien Huo[1], Chih-Ming Chou[2], Wen-Sen Lee[1,3,4*]

[1]Graduate Institute of Medical Sciences, College of Medicine, Taipei Medical University, Taipei 110, Taiwan

Departments of [2]Biochemistry and Molecular Cell Biology and [3]Physiology, School of Medicine, College of Medicine, Taipei Medical University, Taipei 110, Taiwan

[4]Cancer Research Center, Taipei Medical University Hospital, Taipei 110, Taiwan

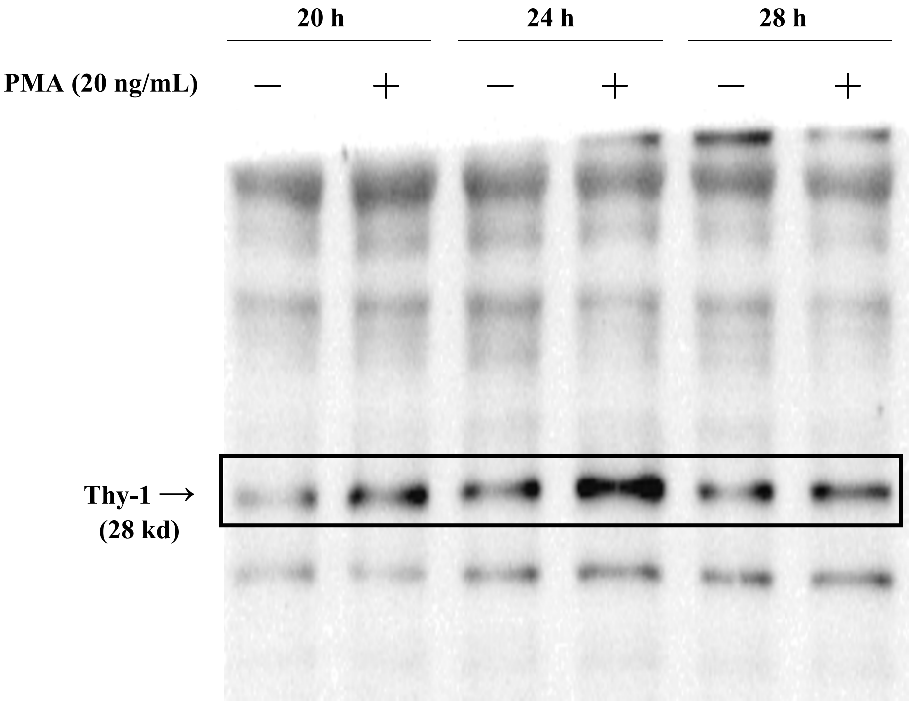

Fig. S1. The entire gel picture of Fig. 1 in the text. Corresponding to Fig. 1B. The cropping line and the molecular weight of the protein were indicated.

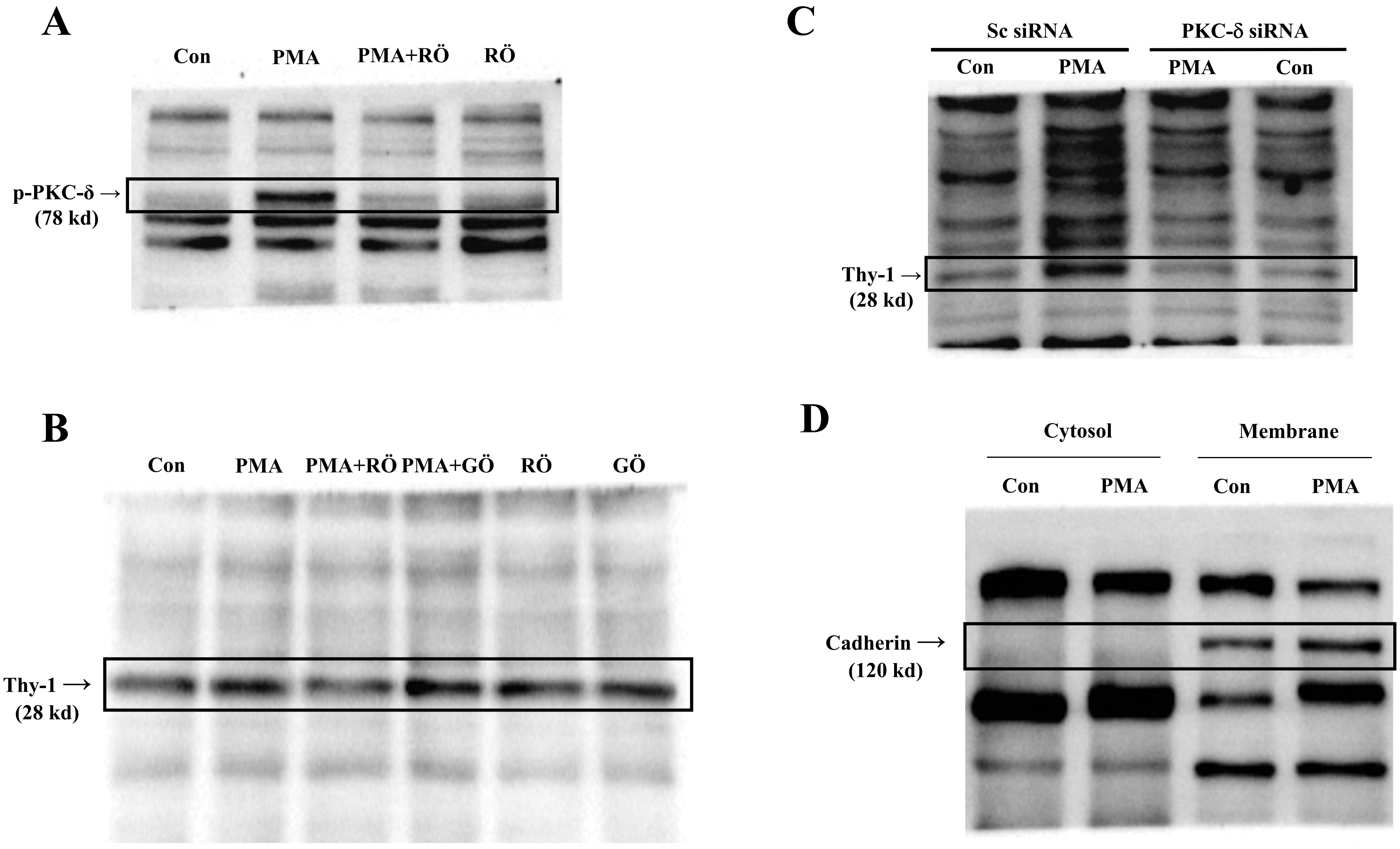

Fig. S2. The entire gel pictures of Fig. 2 in the text. (A) Corresponding to Fig. 2A. (B) Corresponding to Fig. 2B. (C) Corresponding to Fig. 2C (right panel). (D) Corresponding to Fig. 2D (top panel). The cropping lines and the molecular weight of each protein were indicated.

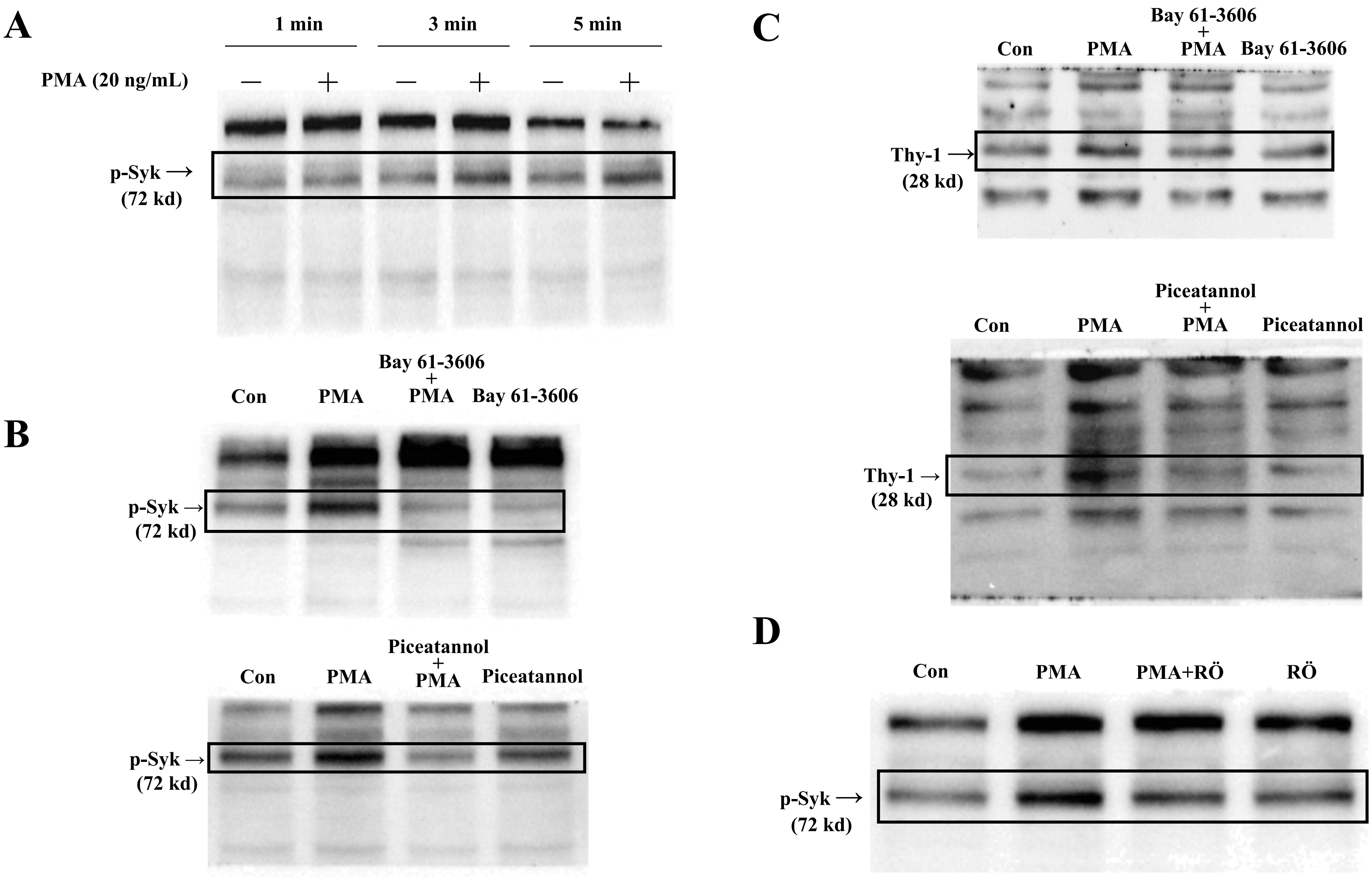

Fig. S3. The entire gel pictures of Fig. 3 in the text. (A) Corresponding to Fig. 3A. (B) Corresponding to Fig. 3B. (C) Corresponding to Fig. 3C. (D) Corresponding to Fig. 3E. The cropping lines and the molecular weight of each protein were indicated.

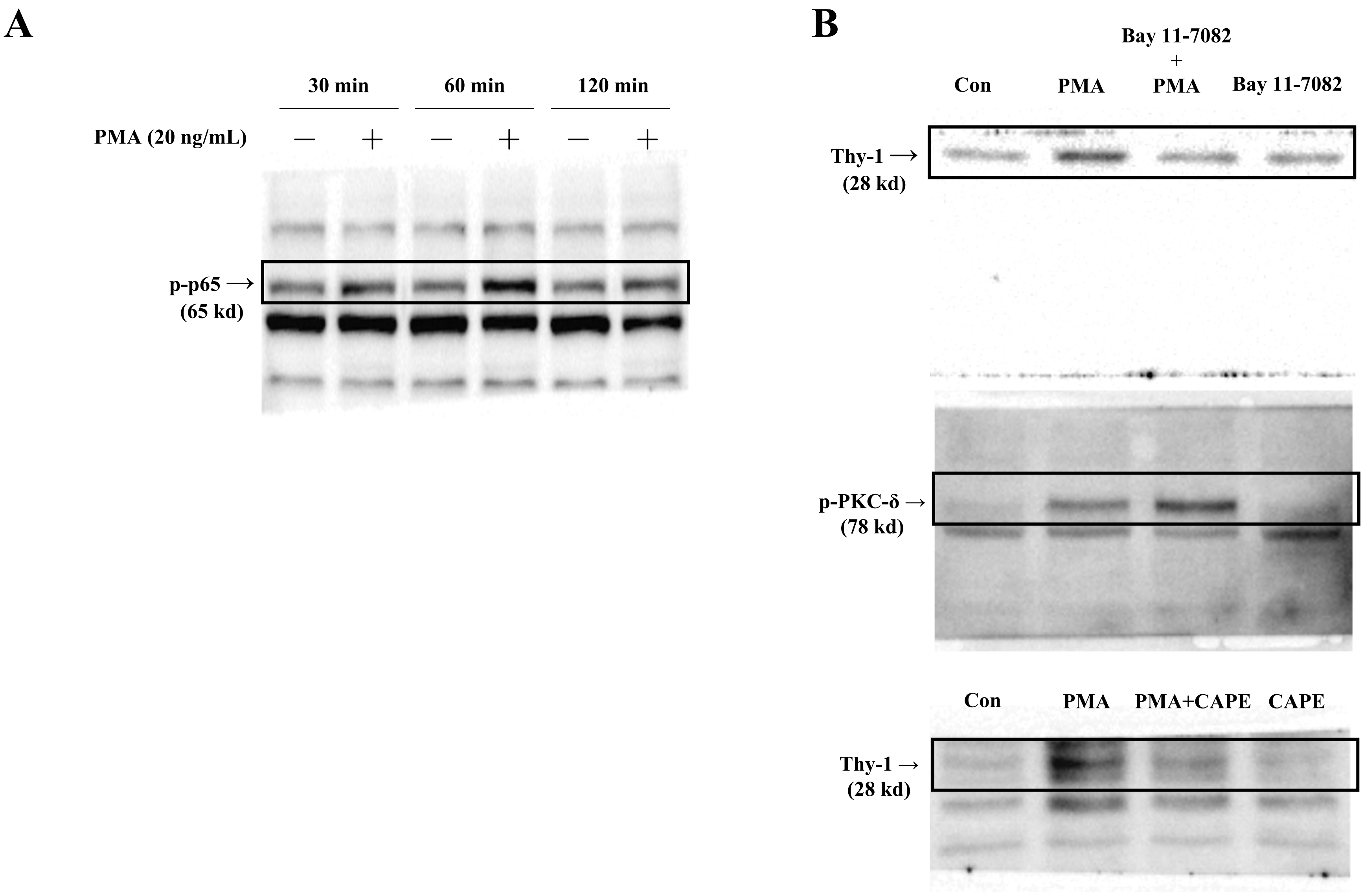

Fig. S4. The entire gel pictures of Fig. 4 in the text. (A) Corresponding to Fig. 4A. (B) Corresponding to Fig. 4E. The cropping lines and the molecular weight of each protein were indicated.

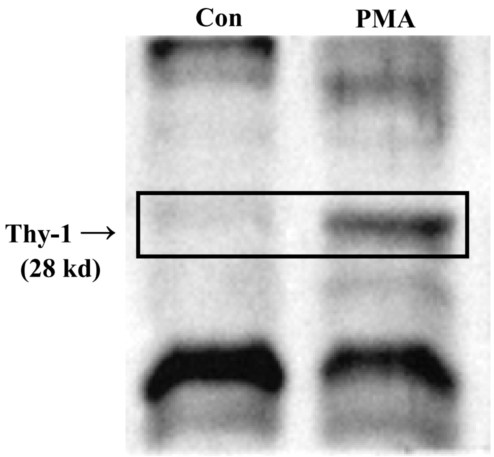

Fig. S5. The entire gel picture of Fig. 5 in the text. Corresponding to Fig. 5B. The cropping line and the molecular weight of the protein were indicated.

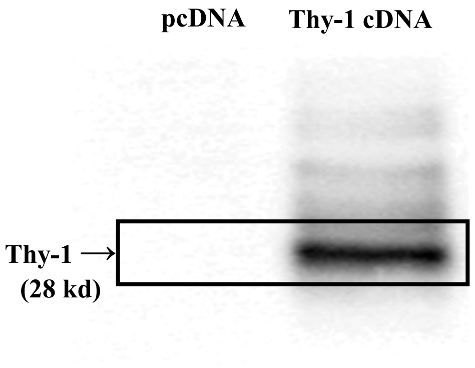

Fig. S6. The entire gel picture of Fig. 6B in the text. Corresponding to Fig. 6B. The cropping line and the molecular weight of the protein were indicated.
